# The BCL‐2 family protein inhibitor ABT‐737 as an additional tool for the treatment of EBV‐associated post‐transplant lymphoproliferative disorders

**DOI:** 10.1002/1878-0261.12759

**Published:** 2020-08-12

**Authors:** Aude Robert, Anaïs Pujals, Loetitia Favre, Justine Debernardi, Joëlle Wiels

**Affiliations:** ^1^ UMR 8126 CNRS Institut Gustave Roussy Université Paris‐Saclay Villejuif France; ^2^ INSERM 1279 Institut Gustave Roussy Université Paris‐Saclay Villejuif 94805 France; ^3^ Département de Pathologie Inserm U955 CHU Henri Mondor Assistance Publique‐Hôpitaux de Paris Université Paris‐Est Créteil Créteil France

**Keywords:** B lymphoproliferative disorders, BCL‐2 inhibitor, drug‐induced cell death, EBV

## Abstract

Post‐transplant lymphoproliferative disorders (PTLD) and Burkitt's lymphoma (BL) are B‐cell malignancies strongly associated with Epstein–Barr virus (EBV) infection. In these lymphoproliferative disorders, EBV infection induces an increase in the expression of the anti‐apoptotic protein BCL‐2. Given its chemoprotective effect, BCL‐2 constitutes an attractive target for new therapeutic strategies for EBV‐positive B‐cell malignancies. Here, we show that ABT‐737, a small inhibitor of BCL‐2, BCL‐X(L), and BCL‐w, strongly induced apoptosis *in vitro* in EBV‐positive lymphoblastoid cell lines (which is a model for PTLD), whereas BL was less sensitive. ABT‐737 reduced tumor growth and increased the overall survival of mice in a xenograft model of PTLD but had no effect on BL xenograft mice. ABT‐737 combined with a low dose of cyclophosphamide, a major component of the conventional CHOP chemotherapy regimen for BL patients, reduced tumor growth during treatment but failed to improve the overall survival of BL xenograft mice. By contrast, the combination of ABT‐737 and rituximab, one of the main options for the treatment of PTLD, was highly efficient and induced approximately 70% remission in PTLD xenograft mice. These results suggest that the use of agents targeting BCL‐2, either alone or in combination with other conventional drugs, represents a novel promising approach for post‐transplant EBV‐positive B lymphoproliferative disorders.

## Graphical abstract

Tumor cells infected by Epstein–Barr virus (EBV) express latency proteins, among which LMP1 is able to increase the level of the anti‐apoptotic protein BCL‐2, today considered as an attractive target for therapeutic strategies. Our results suggest that agents targeting BCL‐2, used either alone or in combination with rituximab, represent a novel promising approach for post‐transplant EBV‐positive B lymphoproliferative disorder treatment.

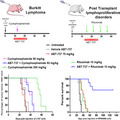

**Abbreviations**
BL: Burkitt's lymphoma
DLBCL: diffuse large B‐cell lymphoma
EBNA: Epstein–Barr nuclear antigen
EBV: Epstein–Barr virus
HED: human equivalent dose
LCL: lymphoblastoid cell lines
LMP: latent membrane proteins
NPC: nasopharyngeal carcinoma
PTLD: post‐transplant lymphoproliferative disorders
SD: standard deviation
SEM: standard error of the mean
TGI: tumor growth inhibition

## 1 Introduction

Epstein–Barr virus (EBV), a ubiquitous B‐lymphotropic herpesvirus, was the first virus directly linked to cancer in humans. Since its discovery, EBV has been associated with a heterogeneous group of epithelial tumors and B‐cell malignancies, including Burkitt's lymphoma (BL) and post‐transplant lymphoproliferative disorders (PTLD). In infected tumor cells, EBV remains latent in an episomal form, with only a small subset of viral proteins expressed, which include six nuclear antigens (EBNA) and three latent membrane proteins (LMP). Differential expression of these latent proteins is observed in EBV‐associated malignancies, which defines three distinct latency profiles. Most EBV‐infected BL cells harbor the latency I phenotype, in which Epstein–Barr nuclear antigen 1 (EBNA1) is the only viral protein produced. Type II latency (expression of EBNA‐1, LMP1, and LMP2) is associated with Hodgkin's lymphoma and nasopharyngeal carcinoma (NPC). In type III latency, typically found in PTLD and some cases of BL, all latent viral proteins are expressed (reviewed in Ref. [1]). The ability of EBV to transform resting B cells into immortalized lymphoblastoid cell lines (LCL) *in vitro* underlies its central role in the pathogenesis of these malignancies.

Burkitt's lymphoma is a rare but highly aggressive non‐Hodgkin B‐cell lymphoma that can be classified into three types based on clinical and epidemiological features. Endemic BL primarily affects children aged 4–7 years in equatorial Africa and Papua New Guinea and accounts for approximately 50% of all pediatric cancers in these areas. Tumors are EBV‐positive in almost every case. By contrast, sporadic BL occurs worldwide and affects both children and adults. Globally, it accounts for 1–2% of adult lymphoma cases. In the United States and Western Europe, it accounts for up to 40% of pediatric lymphoma cases. A third type, called ‘Immunodeficiency‐associated’, is most common in people infected with HIV. It accounts for 30% of non‐Hodgkin lymphoma in HIV patients. Compared to the endemic type, the incidence of EBV infection is considerably lower for these two last types of BL: 10–20% in the sporadic disease, and 30–40% in HIV‐infected patients [2].

Post‐transplant lymphoproliferative disorders are proliferative diseases that develop as a consequence of immunosuppression in 1–2% of patients who receive a solid organ transplant or stem cell allograft. It consists of a heterogeneous group of EBV‐positive or EBV‐negative lymphoid disorders that are indistinguishable from B‐cell or, less often, T‐cell lymphomas that occur in immunocompetent individuals. Nevertheless, the vast majority of PTLD are associated with EBV infection and are monoclonal or occasionally polyclonal [3]. Two studies have reported that both cellular and viral gene expression patterns of PTLD tumors are similar to those of EBV‐infected LCL, suggesting that LCL represents a good *in vitro* model to examine the role of the virus on the specific oncogenic process of PTLD [4, 5].

Significant progress has been made in the treatment of EBV‐associated malignancies, but multiple challenges remain. Thus far, the treatment of BL (intensive chemotherapy, with or without rituximab—a chimeric mouse/human anti‐CD20 monoclonal antibody) is independent of their EBV status and long‐term survival is achieved in 90% of children and approximately 70% of adults [6, 7]. However, the prognosis is poor if patients are refractory to first‐line treatment or they relapse. The treatment of PTLD is not standardized due to its heterogeneity, but usually includes the reduction of immunosuppression, often associated with rituximab and brief low‐dose chemotherapy [8]. Antiviral treatment alone with acyclovir/ganciclovir has doubtful efficacy [9]. Indeed, the prognosis of PTLD depends on its subtype and the timing of occurrence after transplantation but it is generally not good.

The viral proteins that are expressed during latent EBV infection are able to modulate cell signaling and inhibit apoptotic signals, thus promoting survival of the tumor cells [10]. New therapies that target these EBV‐induced cellular properties could therefore constitute additional weapons to treat patients. Among viral proteins, LMP1 is the most likely to be involved in the transforming properties of EBV. LMP1 aggregates at the plasma membrane, where it usurps the function of CD40 and enhances the activation of several signaling pathways involved in oncogenesis, such as the NF‐kappa B, mitogen‐activated protein kinase, and JAK/STAT pathways [11, 12]. One of the oncogenic functions of LMP1 relies on its ability to inhibit apoptosis through the up‐regulation of anti‐apoptotic proteins, such as BCL‐2, A20, and MCL‐1 [13, 14, 15]. Overexpression of BCL‐2 has been found, for example, in LCL and type III BL cell lines [16], as well as in EBV‐positive lymphoproliferative disorders harboring type III latency, including PTLD [17, 18]. Therefore, the use of specific chemical mimetics of BH3 only proteins—which were developed to inhibit the anti‐apoptotic effect of BCL‐2 family members and induce cell death—constitutes a logical strategy to treat EBV‐positive lymphoproliferative disorders.

ABT‐737 was the first BH3 mimetic to be produced [19] and has been tested (as well as navitoclax, its orally bioavailable form) in numerous preclinical models. It efficiently antagonizes various prosurvival members of the BCL‐2 family (BCL‐2, BCL‐xL, BCL‐w, but not MCL‐1 or A1) and increases the susceptibility of various tumor cells to apoptosis (reviewed in Ref. [20]). Here, we report the efficacy of ABT‐737 *in vitro* on EBV‐positive LCL and BL cell lines and *in vivo* on xenograft mouse models. *In vitro*, ABT‐737 strongly induced apoptosis in LCL, whereas BL cell lines were less sensitive, and *in vivo*, ABT‐737 reduced tumor growth and increased the overall survival of LCL xenograft mice (PTLD model) but had no effect on BL models. Furthermore, the combination of ABT‐737 and cyclophosphamide reduced BL tumor growth during treatment but did not improve the overall survival of the mice bearing xenografts. By contrast, the combination of ABT‐737 and rituximab was highly efficient in the PTLD model, as it induced approximately 70% remission. Our results suggest that the use of agents targeting BCL‐2, either alone or in combination with other conventional drugs, represents a novel and promising approach for EBV‐positive B lymphoproliferative disorders, such as PTLD.

## 2 Methods

### 2.1 Cell lines and reagents

The LY47 BL cell line was originally established from an endemic case of BL and kindly provided by the International Agency for Research on Cancer (IARC, Lyon). Seraphina cells were provided by Professor G. Klein (Stockholm). BL2/B95 was generated by stable infection of the original EBV‐negative BL2 (sporadic case of BL) with the B95.8 EBV strain. LCLs were obtained by the *in vitro* immortalization of normal B lymphocytes. The RPMI 8866 cell line was established from the normal B lymphocytes of a 51‐year‐old American woman with chronic myelogenous leukemia (IARC, Lyon). Priess and Remb1 cells were kindly provided by J. G. Bodmer (London). These cell lines were cultured in RPMI 1640 medium (PAA) containing 2 mm l‐glutamine, 1 mm sodium pyruvate, 20 mm glucose, 100 U·mL^−1^ penicillin, and 100 μg·mL^−1^ streptomycin and supplemented with 10% heat‐inactivated fetal bovine serum.

ABT‐737 was kindly provided by Abbott Laboratories (Chicago, IL, USA). Cyclophosphamide (Sigma‐Aldrich, Saint‐Louis, MO, USA), melphalan (Sigma‐Aldrich), and rituximab (Roche, Meylan, France) were reconstituted according to the manufacturer's protocol.

Rabbit anti‐BAX pAb (N‐20), mouse anti‐BCL‐2, and anti‐vinculin were purchased from Santa Cruz Biotechnology Inc (Dallas, TX, USA). Horseradish peroxidase (HRP)‐conjugated donkey anti‐rabbit IgG and HRP‐conjugated goat anti‐mouse IgG used for western blotting were purchased from GE Healthcare (Chicago, IL, USA).

### 2.2 Measurement of cell death

Apoptosis was assessed using the annexin/propidium iodide (PI) assay. We treated 0.5 × 10^6^ cells for 24 h at 37 °C with various concentrations of ABT‐737. Cells were washed in PBS, resuspended in annexin buffer [10 mm HEPES/NaOH (pH 7.4), 150 mm NaCl, 5 mm KCl, 1 mm MgCl_2_, 1.8 mm CaCl_2_] supplemented with 2.5 μg·mL^−1^ FITC‐labeled annexin V (Roche Applied Science, Meylan, France), and incubated at room temperature for 10 min. Cells were then washed, resuspended in annexin buffer supplemented with PI (10 μg·mL^−1^), and analyzed by flow cytometry (*n* = 10 000; FACSCalibur, Becton‐Dickinson, Pont‐de‐Claix, France). Annexin V‐positive cells (PI‐negative or PI‐positive) were considered to be apoptotic.

### 2.3 Preparation of mitochondrial and cytosolic fractions

Aliquots of 2 × 106 cells were resuspended in 100 μL of ice‐cold cell lysis and mitochondria intact (CLAMI) buffer (250 mm sucrose, 70 mm KCl, 200 μg·mL^−1^ digitonin) and incubated at 4 °C for 5 min. The samples were then centrifuged at 1000 ***g*** for 5 min at 4 °C. The supernatants (cytosolic fractions) were recovered and stored at −80 °C. The pellets were resuspended in 50 μL of IP buffer (30 mm Tris/HCL, pH 7.4, 150 mm NaCl, 2 mm EDTA, 2 mm EGTA, 0.2% Triton X‐100, 0.3% NP40, complete protease inhibitor) and incubated for 10 min at 4 °C. Samples were then centrifuged at 10 000 ***g*** for 10 min at 4 °C, and supernatants (mitochondrial fractions) were stored at −80 °C until use in further experiments. Total protein concentration was measured by Bradford assay (Bio‐Rad, Hercules, CA, USA).

### 2.4 Western blot analysis

For each sample, loading buffer was added and the mixture was boiled for 5 min. Equal amounts of proteins were separated by electrophoresis on appropriate Bis–Tris precast gels (LifeTechnologies, Carlsbad, CA, USA) and transferred to PVDF membranes (Millipore, Burlington, MA, USA) by electroblotting. Blots were blocked overnight at 4 °C in 5% nonfat milk powder in PBS and then incubated for 1 h at room temperature or according to the manufacturer's protocol with primary antibodies. After repeated washing, blots were incubated with appropriate HRP‐conjugated secondary antibodies. Antibody complexes were detected with the chemiluminescent HRP substrate (Millipore). The blots were imaged on film (GE Healthcare).

### 2.5 Cellular proliferation assay

Cell viability was assessed using the MTT assay. Cells (2 × 10^5^) were seeded in 200 μL RPMI‐1640 medium into 96‐well plates and treated with ABT‐737, melphalan, or both (triplicate). After 24 h of incubation, 25 μL MTT (Sigma‐Aldrich; 5 mg·mL^−1^) was added to each well, followed by a 2‐h incubation. Then, 150 μL lysis buffer (20% SDS, 45% dimethylformamide, pH 4.7) was added to each well and the plates incubated overnight. Optical density (OD) at 550 nm was measured. The cell viability index was calculated according to the formula: (experimental OD value/control OD value) × 100%. The combination index (CI), which defines synergism (CI < 1), additive effect (CI = 1), or antagonism (CI > 1), was calculated using compusyn software (ComboSyn, Inc., Paramus, NJ, USA).

### 2.6 Ethics statement

This investigation was conducted in accordance with current ethical standards and according to the Declaration of Helsinki, and national and international guidelines were approved by the authors' institutional review board (Gustave Roussy Animal Care and Use Committee). Mice were euthanized under general anesthesia by cervical dislocation when deemed unwell (body weight loss superior to 20% from baseline, tumor mass > 1500 mm^3^, tumor ulceration, dehydration, paralysis, or poor general condition). Necropsy was performed, and tumors were resected when possible.

### 2.7 Subcutaneous xenograft model

For subcutaneous xenografts, LY47 (1.5 × 10^6^) or RPMI8866 cells (2 × 10^6^) were suspended in a 1 : 1 ratio of medium and Matrigel (BD Biosciences, San Jose, CA, USA). Cell suspensions were injected subcutaneously into the left hind flanks of 6‐ to 8‐week‐old mice (PFEP, GR), female NOD/SCID mice (LY47), or NUDE mice (RPMI8866). Mice were randomized according to tumor burden. Drug treatments began between 8 and 12 days following tumor inoculation. Mice were treated daily with 75 mg·kg^−1^ ABT‐737 or vehicle solution for 14 days. A single low dose (50 mg·kg^−1^, equivalent to 150 mg·m^−2^) or high dose (200 mg·kg^−1^, equivalent to 600 mg·m^−2^) of cyclophosphamide was administered intraperitoneally on day 12, or rituximab was administered intraperitoneally once a week (D15, D22, D29, D36) for 4 weeks at 10 mg·kg^−1^. Tumors were measured with a manual caliper every 2–3 days, and volumes were calculated using the formula: tumor volume (mm^3^) = [length (mm) × width^2^ (mm^2^)]/2. Effects on tumor growth were assessed by determining the percentage increase in life span (%ILS), measured as the time for tumors to reach the endpoint: %ILS = [(median time for tumors in the treated group to reach the endpoint/median time for tumors in the control group to reach the endpoint) × 100] − 100; and by determining the percentage of tumor growth inhibition: %TGI = (1 − (mean time for tumors in the treated group to reach size *x*/mean time for tumors in the control group to reach size *x*) × 100.

### 2.8 Disseminated xenograft model

For the disseminated xenograft PTLD model, RPMI8866 cells (10 × 10^6^) were injected intraperitoneally into 6‐ to 8‐week‐old SCID mice (Charles River). The schedule of treatment for ABT‐737 and rituximab was similar to that described above.

### 2.9 Statistical analyses

All numerical data are expressed as means. Data plotted on graphs are expressed as the means ± the standard deviation (SD) or standard error of the mean (SEM). Statistical analyses were performed using two nonparametric tests: the Mann–Whitney test for side‐by‐side comparisons and the Kruskal–Wallis test for multiple comparisons (*n* > 3). Statistical significance for all comparisons was set to *P* < 0.05. The log‐rank (Mantel–Cox) test was performed for the survival analysis using graphpad prism (GraphPad Software, San Diego, CA, USA).

## 3 Results

### 3.1 Evaluation of ABT‐737 efficacy *in vitro*

**Fig. 1:**
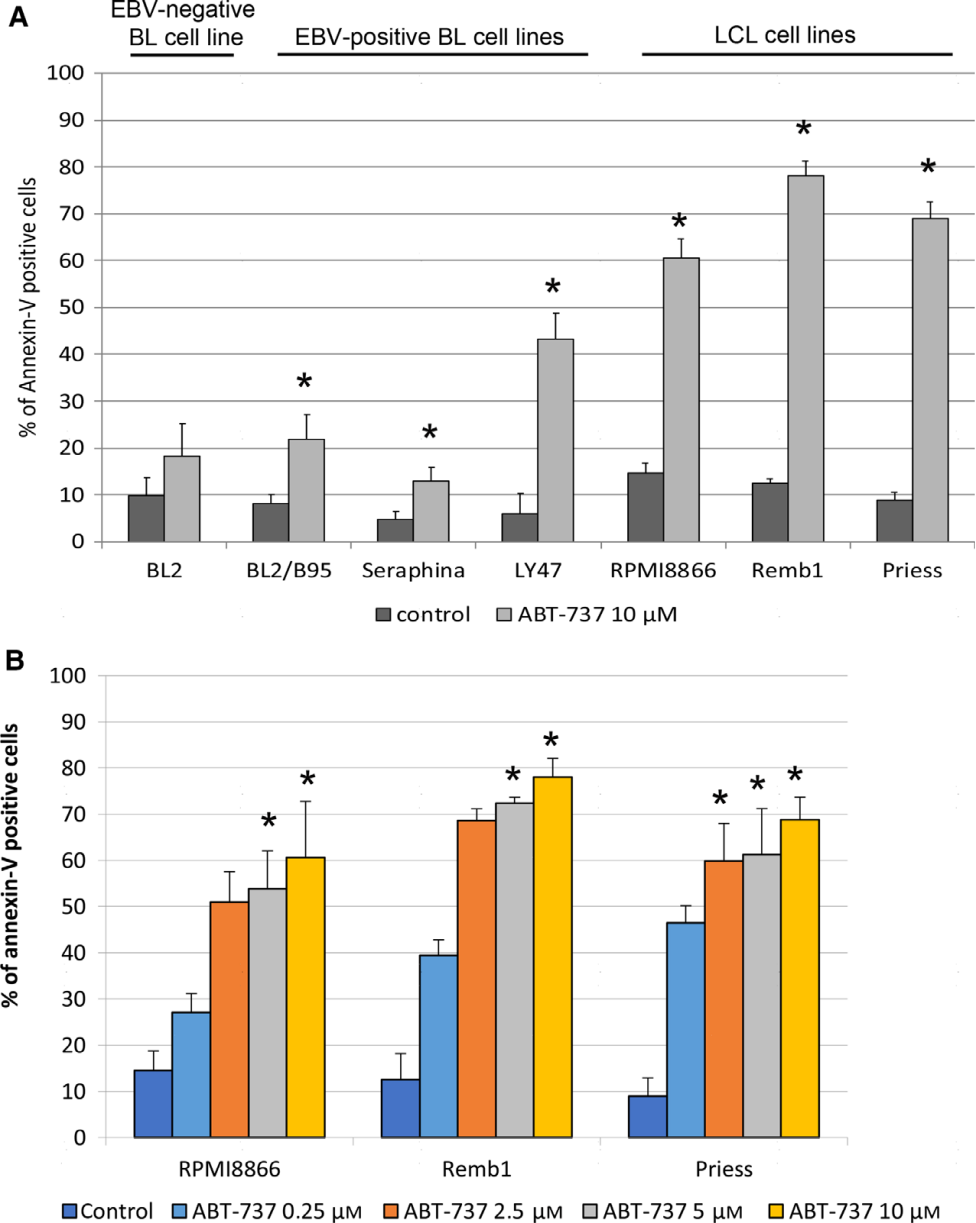
Effect of ABT‐737 treatment on the induction of apoptosis in EBV‐positive B‐cell lines. After treatment, cells were labeled with annexin V‐FITC and PI and analyzed using a FACSCalibur flow cytometer to quantify the percentage of apoptotic cells. The values presented (means ± SD) are from three independent experiments (*n* = 3). (A) EBV‐negative BL, EBV‐positive BL, and LCL cell lines were treated with ABT‐737 (10 µm) for 24 h. Statistical analysis: Mann–Whitney, *P < 0.05. (B) EBV‐positive LCL were treated with various concentrations of ABT‐737 for 24 h. Statistical analysis: Kruskal–Wallis, *P < 0.05.

We first tested the induction of apoptosis by ABT‐737 *in vitro* on an EBV‐negative cell line (BL2) and on a panel of EBV‐positive cell lines, including BL cell lines (BL2/B95, Seraphina, and LY47) and LCLs (RPMI8866, Remb1, and Priess), used as models of PTLD (Fig. 1). Apoptosis was assessed by flow cytometry after labeling the cells with annexin V‐FITC and propidium iodide (PI). Treatment with ABT‐737 for 24 h at a concentration of 10 µm strongly induced apoptosis in LCL, whereas BL cell lines were more resistant (Fig. 1A). Lower concentrations of ABT‐737 were then tested on LCL. Two doses (2.5 and 5 µm) of ABT‐737 were almost as efficient as 10 µm in inducing apoptosis (Fig. 1B).

**Fig. 2:**
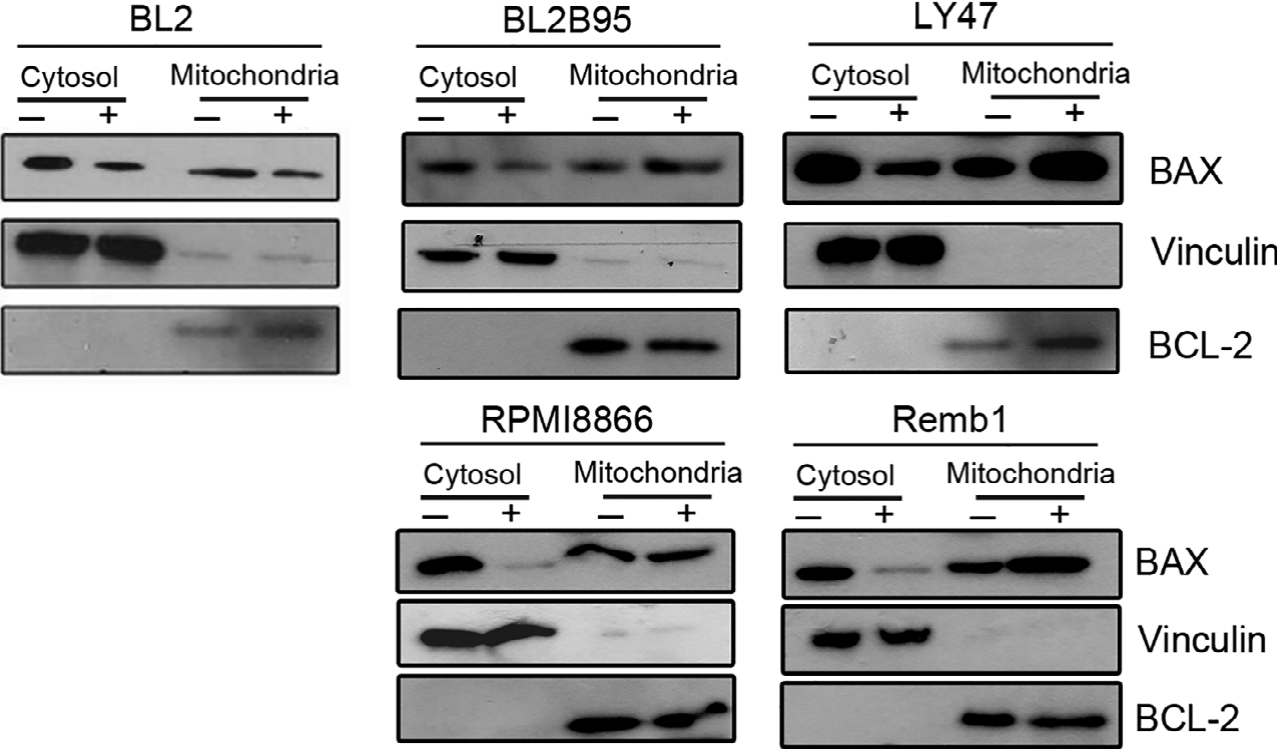
ABT‐737 induced BAX relocalization in EBV‐positive cell lines. BL2 EBV‐negative cell line and BL2/B95, LY47, RPMI886, and Remb1 EBV‐positive cell lines were treated or not with ABT‐737 for 24 h with 2.5 µm (LCL) or 10 µm (BL). Cytosolic and mitochondrial extracts were submitted to western blot analysis for the detection of BAX, vinculin, and BCL‐2 (used as cytosolic and mitochondrial markers, respectively).

ABT‐737 is known to abolish interaction between BAX and BCL‐2 thereby allowing BAX activation and its concomitant translocation from the cytosol to the outer mitochondria membrane. So, the redistribution of BAX was assessed by western blot analysis of the mitochondrial and cytosolic fractions of untreated cells and of cells treated with ABT‐737 for 24 h. Fractionation quality was checked by probing blots with antibodies against proteins known to be localized to the mitochondria (BCL‐2) or the cytosol (vinculin). In the EBV‐negative cell line BL2, treatment with ABT‐737 slightly decreased the amount of BAX in the cytosolic fractions but its level in the mitochondria fractions did not increase. By contrast, ABT‐737 treatment decreased the amount of BAX in the cytosolic fractions and increased its levels in the mitochondria fractions, thereby demonstrating that BAX is activated by ABT‐737 treatment in all EBV‐positive cell lines (Fig. 2).

Many studies have shown that BCL‐2 inhibitors are able to potentiate the effect of conventional treatments in malignant human cells [21, 22]. Cyclophosphamide is a nitrogen mustard alkylating agent, central in the treatment of BL patients (dose 600–800 mg·m^−2^, each cure). However, cyclophosphamide is a prodrug, which needs to be metabolized by liver cytochrome P450 to become an active compound. Thus, for the *in vitro* studies, we used another nitrogen mustard, melphalan, which is a direct alkylating agent. We determined whether ABT‐737 can potentiate the antitumor effect of melphalan *in vitro* by performing MTT assays on the EBV‐positive LY47 BL cell line treated with ABT‐737 (2.5 µm), melphalan (1, 2.5, or 5 µm), or a combination of the two compounds (Fig. S1). At 2.5 µm, ABT‐737 alone showed a minimal cytotoxic effect relative to mock‐treated cells (decrease of 14% in cell viability). When combined with 2.5 or 5 µm melphalan, the combination index (CI) indicated a greater effect than the expected additive effect (CI = 0.21), suggesting that ABT‐737 could be used to reduce the dose of melphalan.

### 3.2 Combination therapy with cyclophosphamide and ABT‐737 in mice engrafted with LY47

First, we performed a dose–response study for cyclophosphamide with concentrations ranging from 25 to 200 mg·kg^−1^. Twenty‐five NOD/SCID mice were injected subcutaneously with LY47 cells. Twelve days after tumor inoculation, cyclophosphamide or its vehicle was administered intraperitoneally. Mice were sacrificed (endpoint) when the tumor volume was > 1500 mm^3^. High doses (100 and 200 mg·kg^−1^) increased mouse survival by 21 and 27 days, respectively, compared to the control group, while low doses (25 and 50 mg·kg^−1^) had moderate effects on survival (increased of 5 and 8.5 days, respectively, Fig. S2). Between 25 and 100 mg·kg^−1^, treated mice presented no side effects, whereas at 200 mg·kg^−1^, mice were prostrated and dehydrated with spiky hair and they lost weight. Moreover, we noticed a temporarily decreased in the number of white blood cells in mice treated with 100 or 200 mg·kg^−1^ (data not shown). We thus decided to use the low dose of 50 mg·kg^−1^ in combination with ABT‐737 and to compare the results to those obtained with 200 mg·kg^−1^ (equivalent to a 600 mg·m^−2^ dose for patient—HED, human equivalent dose, [23]).

**Fig. 3:**
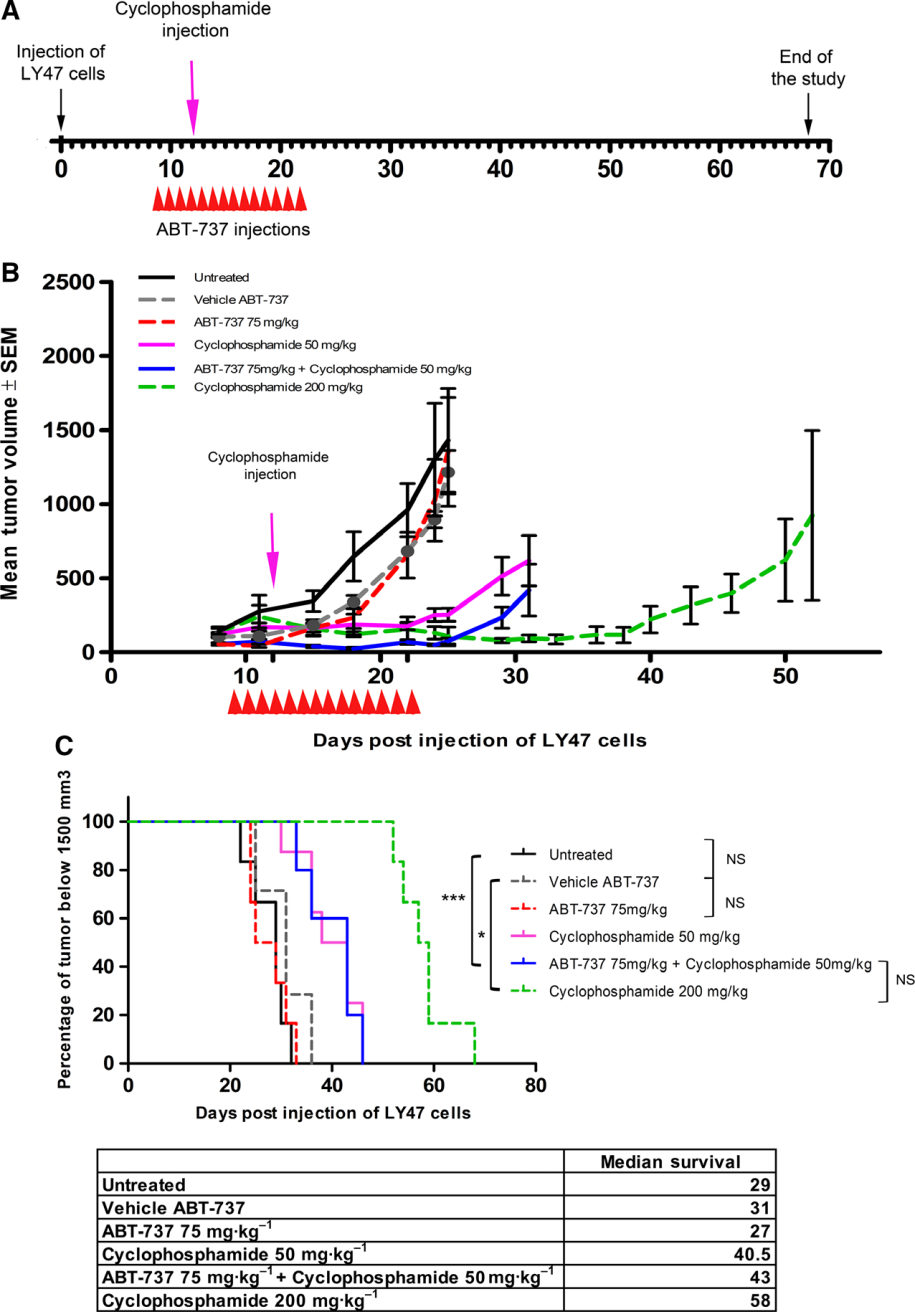
*In vivo* antitumor effect of ABT‐737 in combination with cyclophosphamide in mice with an LY47 cell line‐derived xenograft. Schematic diagram showing timing of tumor implantation and treatment (A), mean tumor volume (±SEM) (B), and Kaplan–Meier plot (C) of mice engrafted with 1.5 × 10^6^ LY47 cells and treated with ABT‐737 (75 mg·kg^−1^·day^−1^ for 14 days; red dashed line, *n* = 6), vehicle (gray dashed line, *n* = 7), ABT‐737 plus low‐dose cyclophosphamide (50 mg·kg^−1^, day 12; blue solid line, *n* = 5), low‐dose cyclophosphamide alone (purple solid line, *n* = 8), high‐dose cyclophosphamide (green dashed line, *n* = 6), or nontreated (black solid line, *n* = 6). Statistical analyses: (B) Kruskal–Wallis, the combination was significantly different from the control groups: *P* < 0.005 at D15, D18, D22, D25. Cyclophosphamide 50 mg·kg^−1^ was significantly different from the control groups: *P* < 0.05 at D22 and D25. Cyclophosphamide 200 mg·kg^−1^ was significantly different from the control groups: *P* < 0.05 at D18, D22, and D25. (C) Log‐rank (Mantel–Cox). *P < 0.05, **P < 0.005, ***P < 0.0005, NS, nonsignificant.

Forty NOD/SCID mice were injected subcutaneously with LY47 cells. Eight days after tumor inoculation, ABT‐737 or its vehicle was administered every day intraperitoneally at 75 mg·kg^−1^ for 14 days (D9 to D22). A single low dose (50 mg·kg^−1^, HED 150 mg·m^−2^) or high dose (200 mg·m^−2^, HED 600 mg·m^−2^) of cyclophosphamide was administered to mice on day 12 (Fig. 3A). Mice were sacrificed (endpoint) when the tumor volume was > 1500 mm^3^.

ABT‐737 alone had no significant antitumor activity [tumor growth inhibition at day 25 [%TGI(D25) = 5%, dashed red line], whereas high‐dose cyclophosphamide (200 mg·kg^−1^, dashed green line) alone induced a strong and long‐lasting inhibition of the tumor growth [%TGI(D25) = 92%]. Low‐dose cyclophosphamide (50 mg·kg^−1^) alone also inhibited the growth of the tumors [%TGI(D25) = 82%, solid purple line], and the combination of the two compounds was more efficient [%TGI(D25) = 95%, solid blue line] (Fig. 3B). However, the addition of ABT‐737 did not extend the effect of the treatment, as the tumor volume in mice treated with both ABT‐737 and cyclophosphamide started to increase shortly after D22. ABT‐737 can potentiate the effect of an alkylating agent *in vitro*. We thus evaluated its efficacy in combination with cyclophosphamide in our *in vivo* BL xenograft murine models.

The effect of the treatments on mouse survival, as measured by the time required to reach the endpoint, is shown in Fig. 3C. Mice receiving saline (solid black line), vehicle (dashed gray line), or ABT‐737 alone (dashed red line) reached the endpoint within 32, 36, and 33 days, respectively. By contrast, mice treated with low‐dose cyclophosphamide (solid purple line) or ABT‐737 + cyclophosphamide (solid blue line) reached the endpoint within 46 days, whereas mice treated with high dose of cyclophosphamide reached the endpoint within 68 days (dashed green line). Treatment with ABT‐737 and cyclophosphamide did not significantly increase survival over low‐dose cyclophosphamide alone, even if there was an increase in life span (ILS) (%ILS = 30.6% for low‐dose cyclophosphamide alone *versus* 38.7% for the combination) and median survival (40.5 *versus* 43 days).

### 3.3 Combination therapy with rituximab and ABT‐737 in mice engrafted with RPMI8866

We performed an additional study to evaluate the *in vivo* effect of ABT‐737 alone or in combination with the chimeric monoclonal anti‐CD20 antibody rituximab in a PTLD mouse model.

**Fig. 4:**
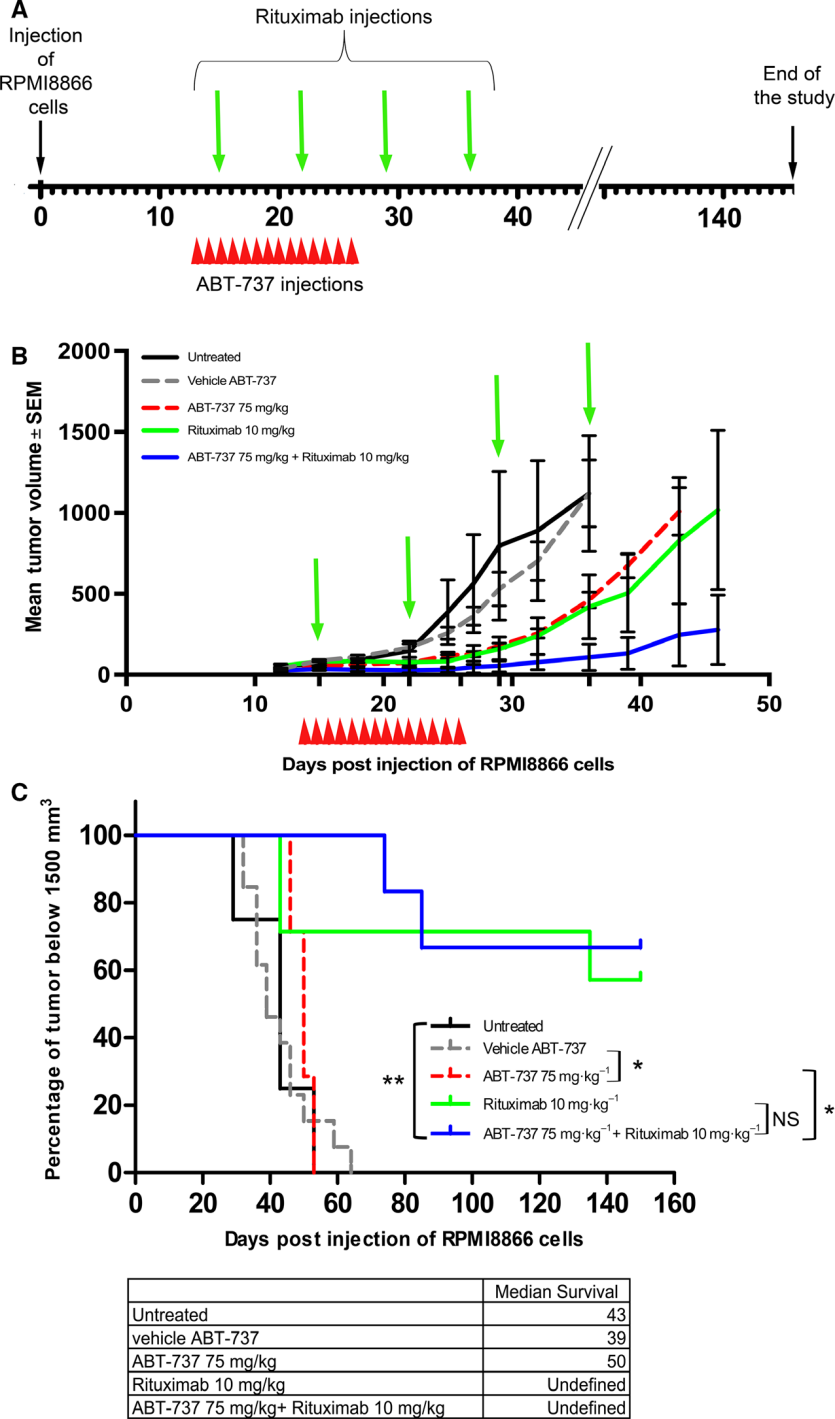
*In vivo* antitumor effect of ABT‐737 in combination with rituximab in mice engrafted with RPMI8866 cells. Schematic diagram showing timing of tumor implantation and treatment (A), mean tumor volume (±SEM) (B) and Kaplan–Meier plot (C) of mice transplanted with 2 × 10^6^ RPMI8866 cells treated with ABT‐737 (75 mg·kg^−1^·day^−1^ for 14 days; red dashed line, *n* = 7), vehicle (gray dashed line, *n* = 13), ABT‐737 plus rituximab (10 mg·kg^−1^; blue solid line, *n* = 7), or low‐dose rituximab alone (green solid line, *n* = 6) or nontreated (black solid line, *n* = 4). Statistical analyses: (B) Kruskal–Wallis, only the combination was significantly different from the control groups: *P* < 0.005 at D18, D22, D25, D29, D32, and D36 and *P* < 0.05 at D12. (C) Log‐rank (Mantel–Cox), *P < 0.05, **P < 0.005, NS, nonsignificant.

A preliminary experiment allowed us to determine that a very low dose of 10 mg·kg^−1^ of rituximab (HED 30 mg·m^−2^, compared to 375 mg·m^−2^ recommended dose) had no side effect on mice but delayed tumor growth (data not shown). Then, 50 nude mice were injected subcutaneously with RPMI8866 cells, and 12 days after inoculation, ABT‐737 (75 mg·kg^−1^) or its vehicle was administered intraperitoneally for 14 consecutive days (D13 to D26). Rituximab (10 mg·kg^−1^) was administered intraperitoneally once a week for 4 weeks (D15, D22, D29, and D36) (Fig. 4A).

The combination of rituximab and ABT‐737 [solid blue line, TGI (D26) = 87%] reduced tumor growth more efficiently than ABT‐737 [dashed red line, TG(D26) = 68%] or rituximab alone [solid green line, TGI(D26) = 78%] (Fig. 4B). ABT‐737 alone was able to slow down tumor growth, as the median survival of this group was longer than that of the untreated group and the group treated with vehicle (50, 43, and 39 days, respectively, %ILS = 28%). However, soon after stopping ABT‐737 treatment, tumor volume markedly increased and all tumors reached the endpoint within 53 days (Fig. 4C), similarly to tumors of the untreated (D53) or vehicle group (D64). The results for the rituximab group were quite heterogeneous, as tumors reached the endpoint at D43 for three of seven mice (43%), tumor growth was delayed in one mouse (14%), and it was completely suppressed in the remaining three animals (43%). When ABT‐737 and rituximab were used in combination, tumor growth was delayed in two of six mice (endpoints reached at D74 and D85) and completely suppressed in the remaining four, as these animals were free of disease at the time of sacrifice (D145).

**Fig. 5:**
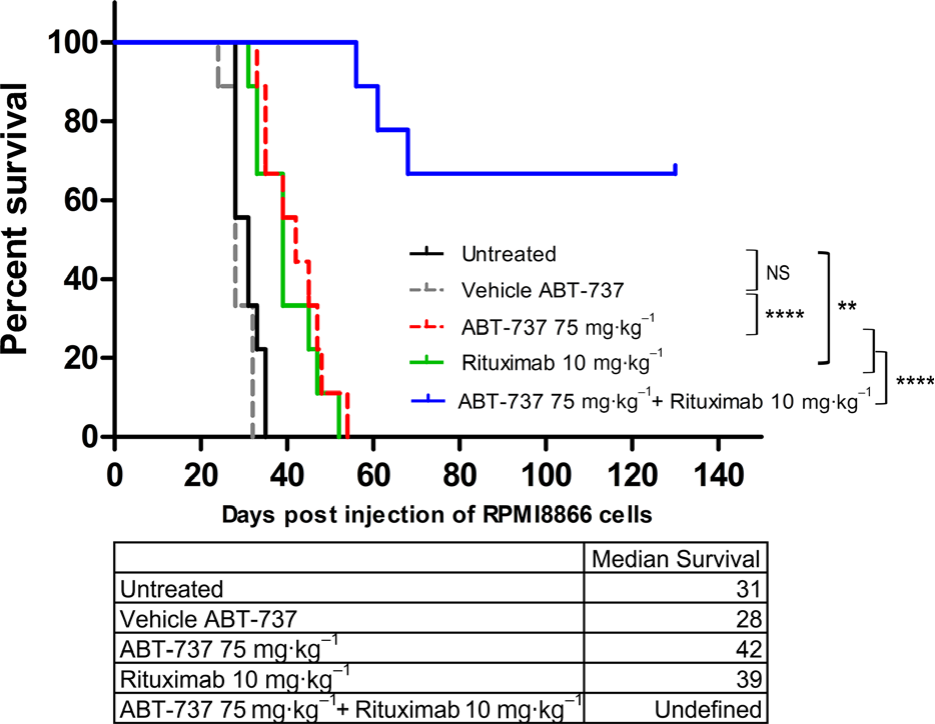
*In vivo* antitumor effect of ABT‐737 and rituximab in the human/SCID chimeric model of PTLD. Kaplan–Meier curves of mice injected intraperitoneally with 10 × 10^6^ RPMI8866 cells treated with ABT‐737 (75 mg·kg^−1^·day^−1^ for 14 days; red dashed line), vehicle (gray dashed line), ABT‐737 plus rituximab (10 mg·kg^−1^; blue solid line), or low‐dose rituximab alone (green solid line) or nontreated (black solid line) (*n* = 9 per arm). Statistical significance was determined by the log‐rank (Mantel–Cox) test. NS, nonsignificant (**P < 0.005, ***P < 0.0005, ****P < 0.00005).

We then more precisely evaluated the overall survival of mice treated with ABT‐737 and rituximab using the human/SCID chimeric model of PTLD. Indeed, it was previously shown that intraperitoneal injection of LCLs results in the reproducible development of a fatal lymphoproliferative disease that develops in the abdominal cavity and viscera 30–50 days postinoculation [24, 25] and shares remarkable similarities with EBV‐positive PTLD in that they are diffuse large B‐cell lymphomas. In these tumors, EBV is uniformly present, with a broad pattern of viral gene expression, and BCL‐2 is abundantly expressed [24, 26]. Fifty SCID mice were injected intraperitoneally with RPMI8866 cells. The schedule of treatment for ABT‐737 and rituximab was similar to that described above. Mice were monitored weekly by ultrasound. Mice were euthanized when abnormal swelling of the abdomen, spiked hair, paralysis, or cardiac rhythm acceleration was observed. Otherwise, the endpoint for the therapeutic trial was death. All animals were subjected to necropsy to determine the growth pattern of tumor development. The overall survival observed for each group is reported in Fig. 5 using a Kaplan–Meier plot.

Treatment with rituximab (green solid line) or ABT‐737 alone (dashed red line) significantly prolonged survival (median survival: 39 and 42 days, respectively) over that of untreated or vehicle‐treated mice (31 and 28 days, respectively). However, all animals treated with rituximab or ABT‐737 (nine per arm) died before D54. By contrast, when ABT‐737 was combined with rituximab (blue solid line), only three mice died (D56, D61, and D68) and six of nine remained tumor‐free until D130 (when they were euthanized). From these experiments, we concluded that ABT‐737 treatment in combination with rituximab can induce complete remission in this PTLD model.

## 4 Discussion

High‐dose combinations of cytotoxic drugs are highly effective in treating BL. However, they are associated with undesirable side effects and, most importantly, often produce adverse health‐related outcomes that occur years after the treatments (late effects). Furthermore, there is currently no standard treatment option for patients who relapse after first‐line therapy and they have a very poor prognosis (https://www.ncbi.nlm.nih.gov/books/NBK65738/). Due to the heterogeneity of PTLD, numerous treatment options have been used (reduction of immunosuppressive drugs, chemotherapy, rituximab, radiation) but the 5‐year overall survival rate is only 20%. Approximately half of patients treated with rituximab are either nonresponders or relapse after an initial complete or partial response [27]. Immunosuppressed and nonresponder patients complicate therapeutic support, and less toxic treatments are needed.

New therapeutic approaches are therefore required, either as alternative treatments or to allow a reduction in the chemotherapy doses. Defects in the apoptotic cascade are a hallmark of cancer and are often associated with chemoresistance. Thus, agents that restore the ability of cancer cells to undergo apoptosis may enhance the activity of chemotherapy when used in combination.

In this context, we performed preclinical studies on BL and PTLD xenograft mouse models aimed at determining the therapeutic potential of inhibiting BCL‐2, either as a monotherapy or as a sensitizing factor for conventional treatments of B lymphoproliferative disorders associated with EBV. Our data show that ABT‐737 in combination with rituximab may represent an effective new treatment option for EBV‐positive and BCL‐2‐expressing post‐transplant lymphoproliferative diseases. Indeed, we show that ABT‐737 alone is as efficient as rituximab in slowing tumor growth and increasing the survival of mice engrafted with LCL. Moreover, in the human/SCID chimeric model of PTLD, ABT‐737 combined with rituximab was the only arm of treatment in which six of nine animals remained tumor‐free for the duration of the follow‐up (130 days). By contrast, ABT‐737 had no effect in the BL model, either on tumor growth or overall survival of the mice. In combination with a low dose of cyclophosphamide, ABT‐737 significantly delayed tumor growth but overall survival of the mice was no longer than that of mice treated with cyclophosphamide alone and much shorter than that of mice treated with a high dose of cyclophosphamide.

Given that both BL cell lines and the LCL used in this study harbor the EBV‐positive latency III phenotype and have similar levels of BCL‐2 [16, 28], it would be informative to better understand their differential responses to ABT‐737 treatment. One possibility may rely on the deregulation of c‐MYC and the PI3K pathway. Indeed, BL cells are characterized by specific translocations that result in c‐MYC activation due to the fusion between the MYC coding sequence and either the immunoglobulin heavy chain locus (t8;14 in ~ 80% of cases) or the immunoglobulin light chain loci (t2;8 and t8;22 for IgK and IgL, respectively, in ~ 20% of cases). Furthermore, recent studies have shown that activation of PI3K signaling cooperates with c‐MYC in the development of BL [29]. Indeed, Spender *et al*. [30] reported that PI3K/AKT or c‐MYC inhibition by specific agents strongly increased ABT‐737‐induced apoptosis in BL cells, suggesting that both the PI3K pathway and c‐MYC are involved in the relative resistance of BL cells to BCL‐2/BCL‐xL inhibition.

Therefore, targeting c‐MYC and/or PI3K in combination with ABT‐737 may be a promising therapeutic strategy to treat BL. Direct targeting of MYC has yet to be achieved but alternatives, such as disruption of the MYC/MAX complex or inhibition of MYC transcription and/or translation, have been explored for the treatment of MYC‐driven tumors (reviewed in Ref. [31]). Since bromodomain and extraterminal (BET) proteins (such as BRD2 and BRD4) regulate MYC‐dependent transcription, various potent and selective small‐molecule inhibitors of these proteins have been developed and clinical trials are currently ongoing in hematological malignancies [32]. A recent study has also already shown that the combination of a BET bromodomain inhibitor (which effectively downregulates MYC levels) with a specific BCL‐2 antagonist (ABT‐199) has synergistic antitumor activity in a xenograft mouse model of diffuse large B‐cell lymphoma (DLBCL) [33]. However, BET inhibitors also interfere with other oncogenic pathways, and the results must be interpreted with caution.

The combination of ABT‐737 and low‐dose cyclophosphamide in MYC and BCL‐2‐deregulated tumors was previously tested by Mason *et al*. They showed that most (14/18) C57BL/6 mice transplanted with Eµmyc/EµBCL‐2 lymphomas and treated with this combination therapy achieved complete remission [34]. Several reasons can account for the discrepancy between this outcome and ours. First, although Eµmyc/EµBCL‐2 murine tumors and EBV‐positive BL cells both overexpress MYC and BCL‐2 proteins, they have very different phenotypes: Eµmyc/EµBCL‐2 tumor cells have a lymphomyeloid progenitor phenotype [35], whereas BL cells have a GC phenotype. The *in vivo* treatment protocols were also quite different between the two models: (a) Mason *et al*. initiated the treatment at D4 following injection of the lymphoma cells, when the tumors were still nonpalpable, whereas we treated randomized animals with established tumors (D8 to 12), and (b) the cyclophosphamide administration schedules were also different, two injections at D5 and D9 for the Eµmyc/EµBCL‐2 model *versus* one injection at D12 in our xenograft model. Given the favorable outcome obtained by Mason *et al* and that treatment with ABT‐737 combined with one injection of cyclophosphamide delayed tumor growth in our BL model, it is possible that repeated (2 or 3) injections of cyclophosphamide combined with ABT‐737 could help to maintain a low tumor burden or even induce complete remission. Such a protocol would also be consistent with standard CHOP treatment, which includes multiple drug cycles.

Due to its physiochemical and pharmaceutical properties, ABT‐737 is not suitable for *in vivo* use in humans and an orally bioavailable analog (ABT‐263/navitoclax) with similar pro‐apoptotic capacity has thus been developed [36]. Although previous *in vivo* studies have shown that both compounds induce thrombocytopenia due to their ability to inhibit BCL‐xL, various clinical trials are currently ongoing (e.g., NCT04041050, NCT00788684, NCT02079740, NCT03181126, and NCT00481091) for both hematopoietic and solid tumors to evaluate the efficacy of ABT‐263/navitoclax (used at appropriate doses to avoid thrombocytopenia) in combination with various drugs. Our results showing the high efficacy of ABT‐737 and rituximab to induce remission in our PTLD xenograft model suggest that therapeutic combination of ABT‐263/navitoclax with rituximab could be used in these patients. In addition, it would certainly be interesting to investigate the effects of ABT‐199/venetoclax, a more specific inhibitor of BCL‐2, in combination with rituximab or cyclophosphamide in BCL‐2 positive lymphoproliferative disorders associated with EBV.

## 5 Conclusions

Tumor cells infected by the oncogenic EBV express latency proteins, among which LMP1 is able to increase the level of various anti‐apoptotic proteins of the BCL‐2 family. This suggests that BH3‐mimetic drugs could constitute new therapeutic agents for EBV‐positive tumors. Our results showing that xenograft PTLD models are sensitive to ABT‐737 (alone or in combination with rituximab) support this possibility. They also suggest that other types of EBV‐positive tumors could be treated by such drugs. Indeed, a recent publication has shown that NPC, an epithelial head and neck cancer associated with EBV that expresses high levels of BCL‐2 in 80% of cases, can be efficiently treated by a combination of ABT‐199 and S63845, an MCL‐1 inhibitor [37]. Patients with NK/T‐cell lymphoma, another type of EBV‐associated disease, could probably also benefit from treatments with BCL‐2 inhibitors, as high expression of BCL‐2 in the tumor cells has been shown to be associated with a poor prognosis [38]. However, no such clinical trials are ongoing. Finally, it is worth mentioning that a subgroup (around 10%) of DLBCL (called EBV‐positive DLBCL not otherwise specified), with poor patient survival, is also associated with EBV [39]. The status of BCL‐2 in this group relative to EBV‐negative cases is still debated, probably because overexpression of BCL‐2 is frequently observed in DLBCL due to genetic mechanisms. With respect to our results on BL cells, various reports have shown that DLBCL that overexpresses BCL‐2 is poorly sensitive to treatment with BCL‐2 inhibitors alone but that combinations with various other drugs are more efficient. Consistent with these observations, a clinical trial was recently initiated to test the effect of adding venetoclax to the usual R‐CHOP treatment (NCT03984448).

## Conflict of interest

The authors declare no conflict of interest.

## Author contributions

AP and AR conceptualized the study, contributed to methodology, formally analyzed the data, and wrote the original draft of the manuscript. LF, JD, AP, and AR validated the data. LF, AP, and AR investigated the data. AR and JW reviewed and edited the manuscript. AR and JW supervised the study. JW acquired the funding. All authors have read and agreed to the published version of the manuscript.

## Supporting information


**Fig. S1.** Effect of ABT‐737 in combination with melphalan on cell proliferation in EBV+ Burkitt lymphoma (BL) cells. LY47 cells were treated, or not, with ABT‐737 (2.5 μM) for 1 h and then left untreated or treated with various doses of Melphalan for 24 h. Cell viability was determined using the MTT assay. The values presented (means ± SD) are from four independent experiments (n = 4).

**Fig. S2.** *In vivo* antitumor effect of cyclophosphamide in mice with LY47 cell line‐derived xenograft. Kaplan–Meier curves of mice transplanted with 2 x 10^6^ LY47 cells treated with 200 mg/kg of cyclophosphamide (green dashed line), 100 mg/kg (red solid line), 50 mg/kg (purple solid line) or 25 mg/kg (blue solid line) or nontreated (black solid line) (n = 5 per arm).

## Data Availability

The raw data are available from the corresponding author upon reasonable request.
