# Supplementary figures and images for: The BCL‐2 family protein inhibitor ABT‐737 as an additional tool for the treatment of EBV‐associated post‐transplant lymphoproliferative disorders
Source: Mol Oncol. 2020 Aug 12;14(10):2520–32. doi: 10.1002/1878-0261.12759 (PMC7530790; doi:10.1002/1878-0261.12759)

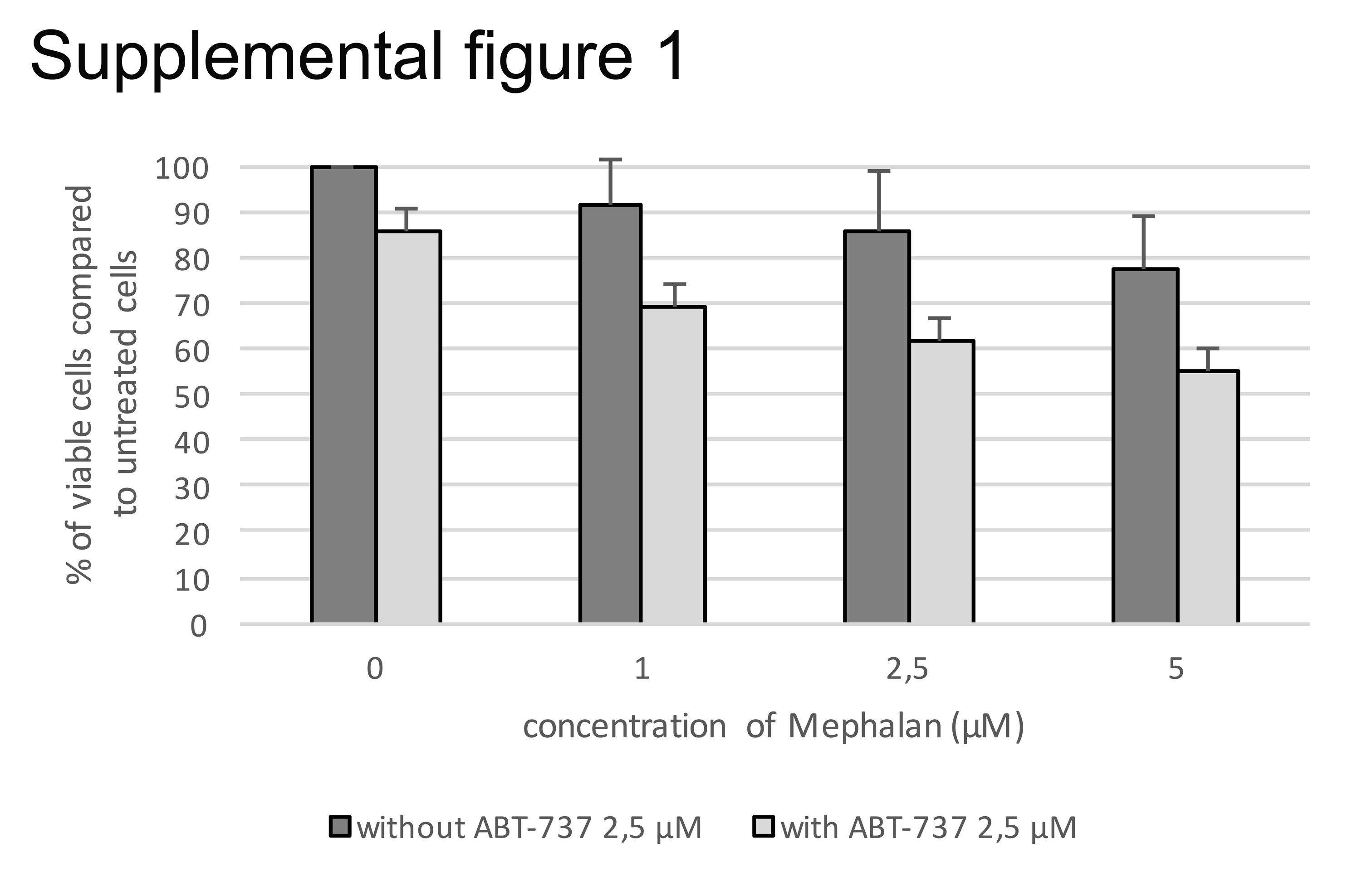

Supplement: Supplementary file 1 — Fig. S1. Effect of ABT‐737 in combination with melphalan on cell proliferation in EBV+ Burkitt lymphoma (BL) cells. LY47 cells were treated, or not, with ABT‐737 (2.5 μM) for 1 h and then left untreated or treated with various doses of Melphalan for 24 h. Cell viability was determined using the MTT assay. The values presented (means ± SD) are from four independent experiments (n = 4). [file MOL2-14-2520-s001.tif]

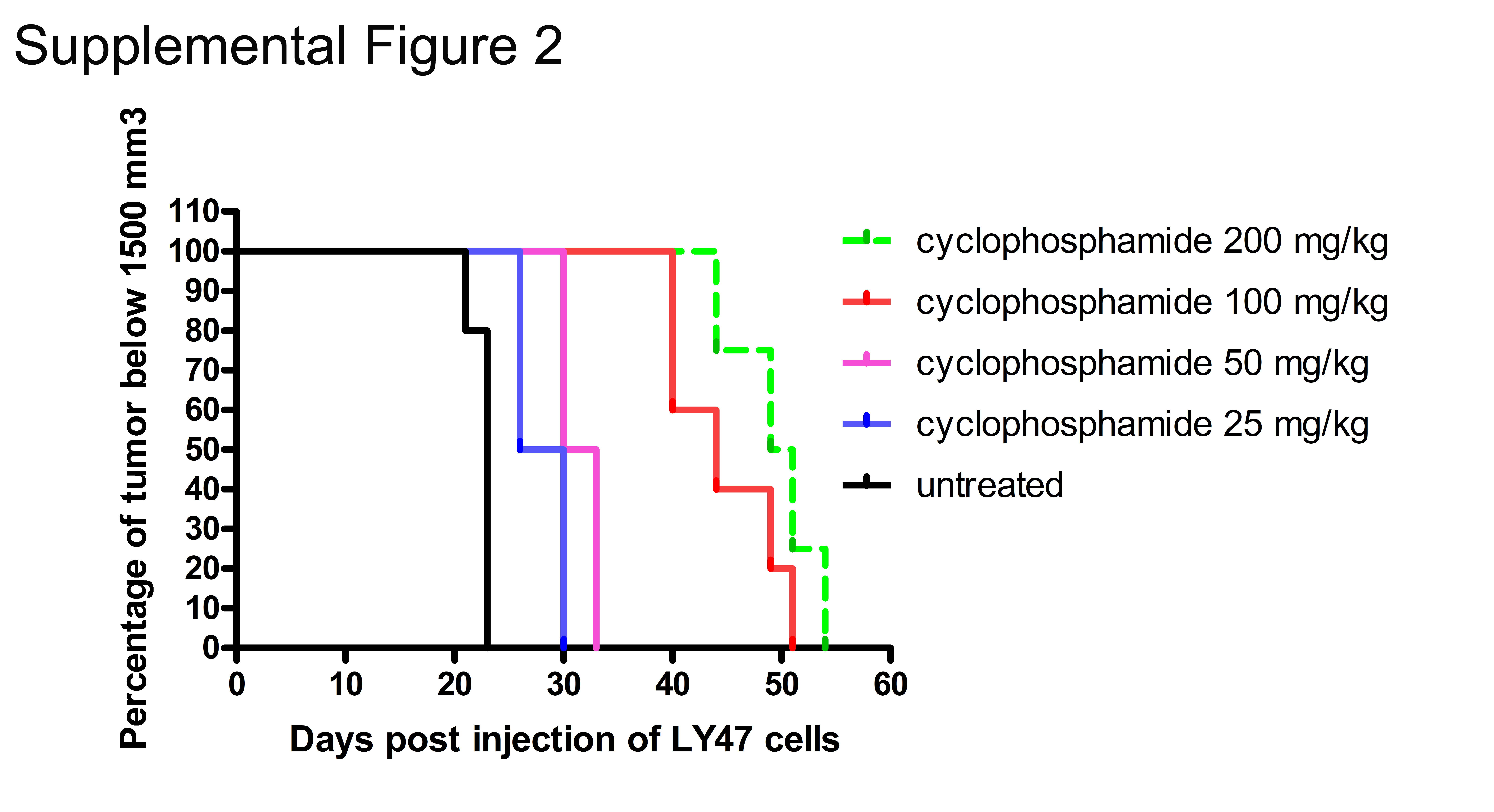

Supplement: Supplementary file 2 — Fig. S2. In vivo antitumor effect of cyclophosphamide in mice with LY47 cell line‐derived xenograft. Kaplan–Meier curves of mice transplanted with 2 x 10^6^ LY47 cells treated with 200 mg/kg of cyclophosphamide (green dashed line), 100 mg/kg (red solid line), 50 mg/kg (purple solid line) or 25 mg/kg (blue solid line) or nontreated (black solid line) (n = 5 per arm). [file MOL2-14-2520-s002.tif]
